# A pilot study testing a continuous glucose monitoring sensor in lean growing pigs fed contrasting diets, to document nocturnal and diurnal glycemic excursions as well as their relationships

**DOI:** 10.1016/j.vas.2026.100612

**Published:** 2026-03-05

**Authors:** Caroline Xavier, Francis Amann Eugenio, Charles-Henri Malbert, Catherine Ollagnier, Sophie Brajon, Florence Gondret

**Affiliations:** a Swine Research Unit, Agroscope, 1725 Posieux, Fribourg, Switzerland; b PEGASE, INRAE, Institut Agro, 35590 Saint Gilles, France; c ANISCAN, INRAE, 35590 Saint Gilles, France

**Keywords:** Continuous glucose monitoring, Diet composition, Nocturnal glycemia, Postprandial metabolism, Precision feeding

## Abstract

Timely non-invasive monitoring of physiology is an emerging field in farm animal research, providing new insights on how animals adapt to environmental challenges and how we can manage performance, health, and welfare. In this study, continuous glucose monitoring (CGM) sensors were used to document glycemic excursions in lean growing pigs. Crossbred pigs (49 ± 3 kg, *n* = 8) were fitted with CGM sensors and indwelling venous catheters, and followed in a five-day feeding trial involving a progressive change in diet composition from high starch (HS) to high fat (HF). At days 2 and 5, time-series blood samples were collected after the first morning meal and used for time-matched chemistry analysis. During the postprandial period, the average CGM reading was higher (*P* = 0.01) on the HS diet than on the HF diet, primarily due to the fact that maximum glucose value at peak was higher (*P* < 0.05) after consuming the HS test meal than the HF meal. During night when the pigs had no access to feed, the period with glucose concentrations above baseline was twice longer (*P* < 0.05) with the HS diet than the HF diet. Various CGM metrics at night were or tended to be correlated with postprandial CGM metrics (*P* < 0.10). Finally, large inter-individual variability was observed in glycemic metrics, larger during day than during the night (*P* < 0.05). To conclude, this study highlights the importance of considering night-time events to inform about animal physiology and further improve precision feeding in pigs.

## Highlights

• Wearable glycemic sensors are informative in lean growing pigs.
• There is a large inter-individual variability in diurnal glycemic excursions.
• Nocturnal glycemia fluctuates with both nadirs and peaks of glucose concentrations.
• Nocturnal glycemic wavelets are influenced by diet composition.
• Nocturnal glycemia events may predict glycemic responses to the first morning meal.

## Implications

Wearable digital sensors could transform the design of physiological and nutritional studies in farm animals. We demonstrated that diurnal and nocturnal glycemic excursions in lean growing pigs varied according to diet composition. Nocturnal glycemic excursions can even predict diurnal metabolic responses to the first morning meal. These data may pave the way for precision feeding technologies for optimizing growth performance, health and welfare of growing pigs.

## Introduction

Growing pigs are usually fed two or three times a day, mimicking their spontaneous feeding rhythm which is reputed to be characterized by two consumption’s peaks, one in the morning and one in the afternoon (Nielsen et al., 1995; Renaudeau, 2009; Andretta et al., 2016). However, new farming technologies such as robotic feed dispensers have recently enabled the tracking of individual consumption, revealing more diverse eating behaviors among pigs (daily meal eaters, nibblers, day-night eaters) and several inconsistencies from day to day (Bus et al., 2023). From a physiological point of view, the misalignment of day-night cycles and feeding rhythm can alter nutrient partitioning and energy metabolism, which can lead to increased fat deposition (van Erp et al., 2020). In the context of feed-food competition and the tremendous pressure on feedstuff supply (crop availability), dietary energy sources can be switched from starch to fat, which is also known to affect metabolism (Gondret et al., 2014). Therefore, the continuous monitoring of physiological events related to nutrition should provide new insights into how pigs adapt to dietary challenges and offers new ways to manage performance, health and welfare. However, this perspective is currently hindered by the lack of wearable sensors for growing pigs.

Circulating glucose is the main energetic nutrient for several organs. Glycemia is tightly controlled but exhibits infra-day fluctuations that may be important for animal nutrition. Indeed, monitoring plasma glucose concentrations over time informs about dietary carbohydrate assimilation (Bach Knudsen et al., 2000), feed efficiency on different diets (Montagne et al., 2014) and how nutrients are used in response to environmental stressors such as heat (Campos et al., 2019; Serviento et al., 2023) and diseases (Merlot et al., 2016). However, this requires an invasive surgery to insert a central catheter on the pig for regular blood sampling. The availability of the staff to collect and process blood samples also limits the frequency of sampling (intervals between sampling points cannot be shorter than 10 min) and the duration of the kinetics studied (a few hours after meal ingestion). Automated blood samplers are available for rodents, but they are not suitable for free moving pigs, due to the small diameter of the connecting tubes. Over the past 15 years, wearable sensors for continuous glucose monitoring (CGM) have been developed in human medicine. These systems include a transmitter unit that adheres to the skin and covers a thin filament extending into the subcutis. They continuously measure interstitial glucose levels and communicate with a handheld reader. Thanks to this monitoring system, infra-day fluctuations in glucose concentrations are now regarded as a risk factor for diabetes-related complications (Fernandes et al., 2022). In healthy people also, CGM may be beneficial. Indeed, an increased number of hyperglycemic and hypoglycemic episodes can lead to protein glycation, oxidative stress and inflammation, which may be harmful to the body (Kohlmoos & Dittmar 2025). Moreover, nocturnal glycemic events have been recently considered for their potential influence on diurnal glycemic features (Huang et al., 2022).

The CGM sensors have already been tested on adult miniature and on conventional pig breeds, diabetic or healthy. When evaluated in a fasting state, a good linear correlation was reported between CGM readings and time-matched glucose concentrations in whole blood samples (Ober & Geist, 2020). Subcutaneous CGM sensors are also suitable for response-time analysis during an intravenous glucose tolerance test (IVGTT) in non-diabetic pigs, with a mean goodness of fit of ∼80 % between CGM sensor data and glucometer measurements (Burnett et al., 2014). In diabetic minipigs, subcutaneous CGM sensors allowed to monitor large amplitudes of glucose excursions after feeding, and spot blood glucose measurements confirmed CGM readings (Strauss et al., 2008). In growing pigs, glycemic excursions have been rarely studied during successive days and nights, even though Lal et al. (2021) documented diurnal and nocturnal events for three pigs with streptozotocin‐induced insulin‐deficient diabetes and showed an overall time‐in‐euglycemic range (70–180 mg/dL) of 58 % between daily meals and of 90 % overnight.

This pilot study aimed to investigate patterns of diurnal and nocturnal glycemic excursions in free moving pigs equipped by CGM sensors and fed different diets over five days. The data revealed the existence of large inter-individual variability in glycemic metrics, which were partly affected by diet composition. We also suggested a relationship between pigs’ capacity to regulate glycemia overnight and responses to daily meals. These data were initially communicated in the abstract form (Xavier et al., 2024).

## Material and methods

### Animals, housing, and feeding

Conventional crossbred ((Large White x Landrace) x Piétrain) female growing pigs were used. From the fattening herd, eight pigs of approximately the same body weight (49 kg ± 3 kg BW) and age (114 *d*± 13 d), were selected within eight different litters and moved into a group pen. Pigs were familiarized to human contact every morning for 5 days. Then, they were successively transferred by pairs to individual pens (0.83×2.28 m) on slatted floor in the same experimental facilities. The ambiance in the room was controlled with temperature maintained at 26 °C and relative humidity at 60 %. A nychthemeral cycle was applied using an artificial neon light and a string of fairy lights between 0800 and 2000 h (12 h daily).

The experiment lasted 2 weeks, including 1 week of recovery from surgery. During the first week, pigs were fed a commercial standard high starch (HS) pelleted diet with a balanced nutrient composition adapted to this stage of growth (Table 1). Thereafter, pigs were enrolled in a 5-days feeding trial. The diet composition was gradually changed from the HS formula to a high fat formula (HF) with 12–13 % lipids (Table 1). Throughout the feeding trial, pigs were fed twice a day (0830 h and 1600 h) with an average daily feed intake of 1 300 g. They had free access to food, except for two days (i.e., day 2 and day 5) when meal test procedures were performed. On d 1 of the feeding trial and in the morning of d 2, pigs received the HS diet. From afternoon distribution of d 2 to d 4, the distributed meals contained decreasing amounts of the HS diet and increasing amounts of the HF diet in the sequence 75/25, 50/50, and 25/75, respectively. At d 5 (morning meal), pigs received only the HF diet. Each pig served as its own control during the trial enabling to consider metabolic responses to diet composition and day-to-day variations. Feed distribution and feed refusals were recorded every day. Pigs had free access to water during the experiment. Pigs were euthanized at the end of the feeding trial just after the last meal test procedure on d 5. A mixture of 25 mg Tiletamine and 25 mg Zolazepam at 15–20 mg/kg BW (Zoletil®, Virbac, Carros, France) was first injected intramuscularly and was followed by intravenous injection of euthanasia agent (T61, INTERVET, Beaucouzé, France; 4 to 6 mL / 50 kg BW).

**Table 1:** Composition of the experimental diets.

Ingredients, % dry matter	HS	HF
Common wheat	39	45
Corn	24	-
Barley	10	-
Wheat bran	4.5	-
Soybean meal	7.0	17
Rapeseed meal	7.0	-
Sunflower meal	3.5	-
Corn starch	-	11
Beet pulp	-	5.1
Vegetable oil	0.4	-
Sugarcane molasse	-	7.0
Lard	-	12
Calcium carbonate	1.0	1.7
Monocalcium phosphate	0.2	0.3
Sodium chloride	0.3	0.4
Sodium bicarbonate	0.6	-
Mineral and vitamin complement	0.5	0.5
Synthetic amino acids	2.0	-
Analyzed chemical composition, %		
Dry matter	87.4	88.3
Crude protein	16.0	13.4
Crude fat	2.4	13.1
NDF	14.8	9.7
ADF	5.3	3.7
ADL	1.59	0.64
Cellulose	4.0	2.9
Starch	46.1	36.5
Ash	4.9	5.7
Nutritional values		
Digestible energy, MJ/kg	13.4	15.6
Net energy (NE), MJ/kg	9.7	11.8
Digestible Lys / Net energy	1.05	0.47

Abbreviations: HS = High starch diet; HF= High fat diet; NDF = Neutral detergent fiber; ADF = Acid detergent fiber; ADL = Acid detergent lignin.

### Sensor device and analysis of readings

At the beginning of the experiment, each pig was surgically fitted with a jugular silicon catheter under general anesthesia. After an overnight fast, the pigs were pre-anesthetized with an intramuscular injection of 15 mg/kg BW of a mixture of 25 mg Tiletamine and 25 mg Zolazepam (15–20 mg/kg BW, Zoletil®). Anesthesia was achieved with isoflurane (Isoflurane, Baxter, Maurepas, France, 3 % v/v) administered using a gas mask using enriched air with oxygen (40 % v/v). An indwelling silicone catheter was inserted through a collateral vein of the right external jugular and externalized through the back of the pig’s neck. The pig was returned to its individual pen before it regained consciousness. Analgesia (0.4 mg/kg BW of Meloven® or 3.0 mg/kg BW of ketoprofen) was ensured during 2 days. Feed was reintroduced the same day, and feed allowance was then gradually increased for the next 3 days after surgery. The wound was daily cleaned and disinfected using an PVB iodine-based solution (Betadine®). On the second and fourth day after surgery, 1 mL per 10 kg BW of amoxicillin (Duphamox, Zoetis, Malakoff, France) was injected. Catheters were flushed every two days with a 5 mL saline solution mixed with heparin (1 %). A pain assessment grid including four types of criteria (posture, behavior, appearance and sensitivity of the wound) was completed for each pig during the recovery period, and rectal temperature was also measured every day. The pig was fasted again the night before the start of the feeding trial. At d 1 of the feeding trial, each pig was tranquilized for a maximum of 15 min using the pre-anesthetic cocktail (Zoletil®). Then, a small skin area close to the neck was shaved and a light asepsis was performed using alcoholic chlorhexidine mixture (chlorhexidine / benzalkonium/ benzylic alcohol.0.25 g /0.025 *g*/ 4 mL for 100 mL) This mixture was preferred to iodine-based mixture since the strong oxidative properties of iodine could interfere with the sensing mechanism of the CGM device (i.e., the glucose oxidase cyclic amperometry measurement). Different locations for CGM sensor insertion (leg, belly, neck) were tested prior to this experiment. The neck area was chosen because it provided the most consistent readings and it was where the pig was least likely to tear off the sensor. The CGM sensor (Dexcom® G6, Dexcom Inc., San Diego, California) was placed using the applicator provided by the supplier and secured with an adhesive patch attached to the sensor. The low-energy (LE) blue-tooth transmitter was connected immediately to the sensor and was placed in the preparation mode that lasted 2 h according to the manufacturer’s instructions. The user cannot obtain any reliable measurements until the system unlocked automatically after the completion of this time period. The entire sensor, including the transmitter, was secured using strengthened elastic bands (Tensoplast Vet 8 cm wide) around pig’s neck. A surgical swab was inserted between the sensor and the adhesive band, which allowed the sensor having some degree of movement while the pig was moving, preventing dislodging. The LE Bluetooth receiver was then electronically paired to a dedicated receiver (Dexcom receiver PN MT24078–1, software revision 5.1.2.035). The receiver was hung in the center of the pen to ensure a transmitter/receiver distance of <6 m It was permanently connected to the mains, which prevented battery drainage during the extensive recording periods. Immediately before the pairing, the receptor was reset to factory settings to ensure that no previous measurements remained. Prior to pairing, the exact time obtained from a web-based atomic clock (https://www.lne.fr) was set in the receiver and served as a reference for the entire experiment including manual blood sampling. At this time, a small volume of blood (∼400 µL) was collected through the jugular catheter and used to assess the hematocrit (Micro hematocrit centrifuge Servospin SMART, Servoprax GmbH, Wessel, Germany). Glucose concentrations were automatically recorded every 5 min by the CGM sensor and stored in the receiver internal memory. At the completion of the recording session, the receiver was attached to a computer (OSX 13) running a python 3 open-source software Dexcom Reader (https://github.com/openaps/dexcom_reader), that serves to upload the raw data (unfiltered data) as a csv file. Each glucose data point was associated with a unique time-tag corresponding to the internal clock of the receiver. Data that were considered off-scale by the receiver were indicated as NaN. They were further processed according to Piersanti et al. (2023).

After the experiment, the entire CGM file was first analyzed for calculation of MAGE (mean amplitude of glycemic excursions) and CONGA (continuous overall net glycemic action: 1 h, 2 h and 6 h), which are two well-established metrics of glycemic fluctuations in humans (e.g.; Köhlmoos & Dittmar 2025). This was achieved using the QoCGM open-source software (https://github.com/simcich/QoCGM) implemented in MATLAB R2024b. The MAGE and CONGA calculation algorithms are described in Cichosz et al. (2025) who also gives the actual mathematical formulas in the appendix. Results of these calculations for the experimental pigs are illustrated in Supplementary Figure S1.

The same csv file was then filtered to remove missing data (i.e., those assigned as NaN by the receiver), and smoothed according to a Savitsky-Golay filtering (Sadıkoglu & Kavalcıoğlu, 2016) which is classically used within the scope of CGM measurements, using a home-designed software written under Labview 2024 (National instruments, Texas). Recursive interpolation was used to remove single missing values. When two or more missing data or erroneous values are identified, the system recreates the missing sections using Piecewise Cubic Hermite Interpolating Polynomial (Rabbath & Corriveau, 2019). While this failsafe mechanism exists, it was never triggered in our experiment since missing data were extremely rare and limited to one sample only. The parameters of the Savitsky-Golay filter were identical to those described by Yadav et al. (2020) and were primarily set to achieve denoising of the incoming signal. Ultimately, the software created a text file that was suitable for R statistical analysis.

#### Meal tests and serial blood sampling

Two meal test procedures were conducted on d 2 and d 5 of the feeding trial. Feed was removed at 1700 h on the preceding night (i.e., night 1 and night 4). On the next morning, a first blood sample was collected by the jugular catheter at 0845 h. At 0900 h, a calibrated portion of either the HS diet (d 2) or the HF diet (d 5) was given. This calibrated portion (approximately 470 g) corresponded to 70 % of the pig's spontaneous consumption of the previous morning meals, ensuring that it was eaten in <10 min (Le Floc'h et al., 2019). Blood was then collected at 10, 20, 30, 40, 50, 60, 75, 90, 105, 120, 150, 180, 210 and 240 min postprandially. At each sampling, 2 mL of blood was drawn through the catheter and transferred into heparin coated tubes. The tubes were immediately placed on an ice bed, and plasma was rapidly prepared by centrifugation (3 000 x *g* for 10 min at 4 °C) and stored at −20 °C until analyses. The catheter was flushed with sterile saline after each sampling. After the last blood collection, at 1330 h, pigs received the dietary complement of the morning meal; the afternoon meal was given at 1700 h.

Plasma concentrations of glucose were determined in duplicate with a commercially available kit using colorimetric methods (Glucose HK, Thermo Fisher Scientific, Vantaa, Finland) and an automatic analyzer Konelab 20i (Thermo Fisher Scientific, Courtaboeuf, France).

### Glycemic excursions statistics

Filtered CGM data were first used for Bland Altman comparisons between CGM readings and circulating glucose concentrations obtained from blood sampling. This analysis was primarily performed in the Bland Altman module of Prism 10 (GraphPad, USA). The bias and the lower and upper 95 % limits of agreement were extremely large (Supplementary Figure S2). This did not actually indicate a fault with the CGM, but was rather the consequence of a longer delay between the CGM reading and the actual change in blood glucose in pigs compared to humans, for whom a delay of 9.5 min is observed (Schmelzeisen-Redeker et al., 2015). Indeed, unlike humans, porcine erythrocytes are not permeable to glucose (Kim & Luthra, 1977). Therefore, the CGM absolute values could not directly reflect plasma glucose in pigs. We developed a Labview software based on the insights from type 2 diabetes (Sinha et al., 2017; Kulcu et al., 2003), based on frequency-domain cross-correlation analysis to extract accurate time-shift value between CGM and blood-borne values. As the temporal resolution of the CGM signals is 5 min (i.e., too small for high-resolution cross-correlation analysis due to the inherent limitations of the Nyquist criterion), we performed spline interpolation to obtain 1-min intervals between consecutive blood glucose and CGM data points. The resulting matrix was cross-correlated, and the peak of the cross-correlation function was identified. Its index value was converted into a time difference. This value was used to shift the incoming CGM value, with padding values equal to the first (or last) CGM value of the sequence. The plasma glucose was converted to blood glucose by applying the hematocrit value as a conversion factor. Ultimately, the converted CGM data and the blood data were submitted to Bland-Altman analysis. The results of this analysis are shown in Supplementary Figure S2, indicating only small bias.

To analyze the glycemic excursion, the night period was defined from 2100 h to 0655 h, while the diurnal period was defined from 0700 h to 2055 h. At d 2 and d 5 of the feeding trial, the postprandial period was defined from 0845 h (to include the first sampling time at 15 min before meal distribution) to 1300 h (end of the test meal procedure). The profiles of CGM readings resemble mountain landscapes, and the metaphors "peaks” and “nadirs" were used to describe sharp rises and falls in glucose levels, respectively (e.g.; Kramer et al., 2014). All analyses were done in R version 4.2.2 (R Core Team, 2022). The baseline for circulating glucose concentrations of each pig was defined as the mean plus or minus one standard deviation (SD) of the its following day. Values above the baseline (respectively, below) were grouped to define peaks (respectively, nadirs). Each excursion was described by its maximum value for peak (respectively, its minimum value for nadir), the duration (interval calculated in minutes between the first and last values above or below the baseline) and the area under the curve (AUC) which was computed with the “lintegrate” function (Hallman, 2021). For each period (night, postprandial, day), the sum of the duration and the sum of the AUC of the peaks (respectively, of the nadirs) were also calculated. We counted the peaks and nadirs in each period. The most pronounced peak (respectively, nadir) was selected as having the highest value (respectively, the lowest value) for glucose, and its associated characteristics (AUC, duration) were computed. For statistics, the pig was the experimental unit. Pearson correlation coefficients (r) were calculated between the CGM data and catheter-obtained plasma concentrations of glucose at the same sampling time, by using the “rmcorr” function (Bakdash & Marusich, 2024) to account for paired measurements. One-way ANOVA was performed to test the effect of diet composition (HS or HF) on CGM metrics, by using the “Anova” function (Fox & Weisberg, 2019). Spearman correlations (ρ) were calculated between glycemic excursions during the postprandial period and glycemic excursions in the former night, by using the “cor.test” function. Glycemic excursions between the night and the day (i.e., for night 2 / day 3 when no meal test procedure had influenced the spontaneous feeding behavior of the pigs) were compared for their descriptive CGM metrics, by using an Anova Type 3 (Fox & Weisberg, 2019). The variability of each CGM metrics in these periods was compared with F test (R Core Team, 2022). Finally, a principal component analysis (PCA) was performed to provide an overview of the correlation matrix and facilitate graphical interpretation of the relationships between glucose excursions at night and in the day, by using the “pca” function (Lê et al., 2008). In all analyses, *P*≤ 0.05 was considered significant, and 0.05 < *P*≤ 0.10 was discussed as a trend.

## Results

During the experiment, no particular health problems were observed, and spontaneous feed consumption was within what is expected for pigs at this stage of their growth.

### Comparisons between CGM readings and catheter-obtained plasma glucose concentrations

**Fig. 1:**
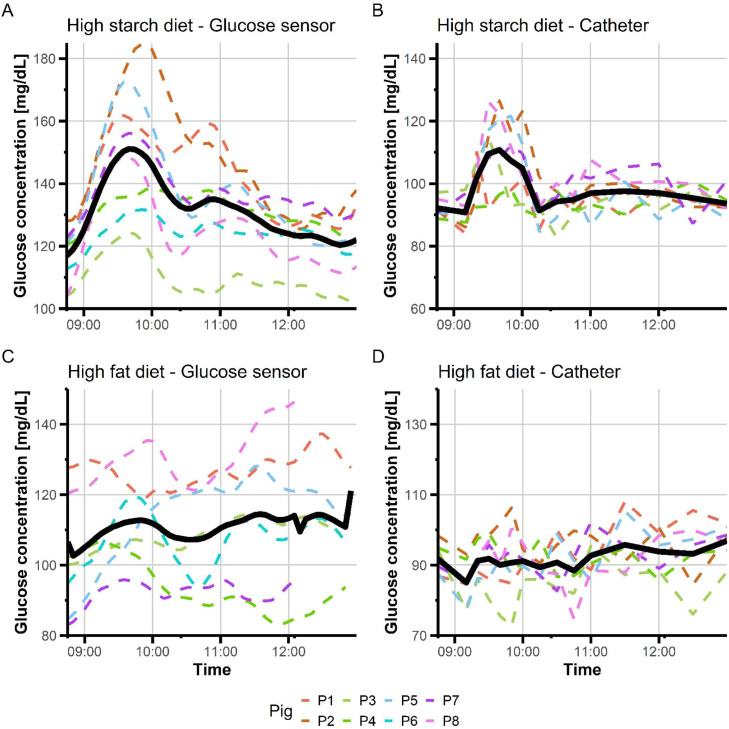
Postprandial variations in glucose concentrations in growing pigs. Time series variations in glucose concentrations just before (15 min) and after (during 3 h eating a test meal rich in starch (A, B: HS, *n* = 6 pigs) or fat (C, D: HF, *n* = 7 pigs) were read from interstitial fluid using a continuous glucose monitoring sensor (CGM) (A, C) and measured by chemical kits in plasma from blood sampled via a jugular catheter (B, D).

One pig ripped off the CGM sensor after one day of use (i.e., the day of the HS meal test); the sensor was replaced and used for the last night of the feeding trial (night 4) and the following day (d 5 of the HF meal test). A second CGM sensor stopped working at the beginning of d 5. With the glycemic baseline established the night before, the conditions for defining peaks after eating the HS test meal were not met in two pigs which were therefore excluded from the calculations. This deviation of the CGM readings during the first two days after insertion have been also noticed by Kvist et al. (2006) working on hyperglycemic pigs. Finally, a catheter stopped working for d 5. As a result, a total of six pigs and seven pigs with complete CGM data and their matched catheter blood samples were considered for studying glycemic excursions in the postprandial period on the HS diet (d 2) or the HF diet (d 5), respectively. Fig. 1 shows the glycemic excursions during the first three hours after eating the morning meal test, as recorded by CGM or serial plasma analyses. The CGM readings and plasma glucose concentrations during the postprandial periods were significantly correlated (*P*< 0.01; Supplementary Figure S2), but the correlation was much higher on the HS diet (*r*= 0.70) than on the HF diet (*r*= 0.43). Time-adjustment correction was performed using frequency-domain cross-correlation analysis to produce Bland Altman comparison (Supplementary Figure S2). This suggests that there was only a slight bias in the corrected CGM readings compared to the time-matched blood glucose concentrations. The time difference between the occurrence of the peaks was 14 min on average. Overall, CGM readings overestimated plasma glucose concentrations, especially during the period after the first peak in the morning (Fig. 1): +37 mg/dL on average when pigs were fed the HS diet, and +19 mg/dL on average when pigs were fed the HF diet.

#### Dietary effects on glycemic excursions as measured by CGM sensors

The MAGE and CONGA were constantly lower for the HF diet compared to the HS diet, but the difference did not reach statistical significance (Supplementary Figure S1). During the morning period (Fig. 1), the average of CGM readings was higher (*P*= 0.01) with the HS diet (132.7 mg/dL ± 16.3 mg/dL) than with the HF diet (111.3 mg/dL ± 15.0 mg/dL). The peak of glucose value read by the CGM sensors was reached between 35 and 55 min after eating the HS meal, and between 20 and 155 min after eating the HF meal, respectively. The maximum glucose concentration at peak was different (*P*< 0.05) between diets, with 159 mg/dL for the HS diet and 132 mg/dL for the HF diet, respectively. There were no differences in other CGM metrics during the three hours following ingestion of the test meal between HS and HF diets (Table 2).

**Table 2:** Effect of diet composition on glycemic excursions.

	Diets			
CGM metrics	HS^1^	HF	SEM	*P*-value
Postprandial peaks				
Number (n)	1.4	0.8	0.7	0.22
Total duration (min)	64	90	66	0.61
Total AUC	660	1 010	1 074	0.67
Maximal glucose concentration (mg/dL)	154	128	14	0.04
Duration of the most pronounced peak (min)	43	85	58	0.36
AUC of the most pronounced peak	545	1 002	1 028	0.56
Postprandial nadirs				
Number (n)	0.8	0.8	0.9	1.00
Total duration (min)	73	85	75	0.87
Total AUC	364	446	567	0.88
Minimal glucose concentration (mg/dL)	122	106	18	0.38
Duration of the most pronounced nadir (min)	33	68	6	0.53
AUC of the most pronounced nadir	195	370	481	0.72
Nocturnal peaks				
Number (n)	1.5	2.3	0.7	0.07
Total duration (min)	87	76	21	0.39
Total AUC	599	689	406	0.71
Maximal glucose concentration (mg/dL)	152	139	19	0.26
Duration of the most pronounced peak (min)	76	43	19	0.01
AUC of the most pronounced peak	562	562	421	0.99
Nocturnal nadirs				
Number (n)	1.8	2.0	0.9	0.76
Total duration (min)	76	64	35	0.57
Total AUC	326	592	298	0.17
Minimal glucose concentration (mg/dL)	112	84	23	0.06
Duration of the most pronounced nadir (min)	70	37	28	0.06
AUC of the most pronounced nadir	316	466	223	0.29

Abbreviations: AUC = area under the curve; HS = High starch diet; HF = High fat diet.

1 Data were recorded the night and the following morning when a meal test procedure composed of the HS diet (day 2, *n* = 6) or the HF diet (day 5, *n* = 7) was performed. Pigs were prevented from accessing the feed from 1600 the night before. The night data were computed from 2100 to 0655. The postprandial period was defined as the interval starting 15 min before and lasting for 3 h following the distribution of the morning meal test (end at 1300 h). The daily blood glucose profiles obtained from the CGM sensors resemble mountain landscapes, leading to the use of metaphors "peak and nadir" to describe sharp rises and falls in blood glucose levels. Duration was defined as the time interval during which glucose values remained above or below the baseline within successive serial data for peak or nadir, respectively.

**Fig. 2:**
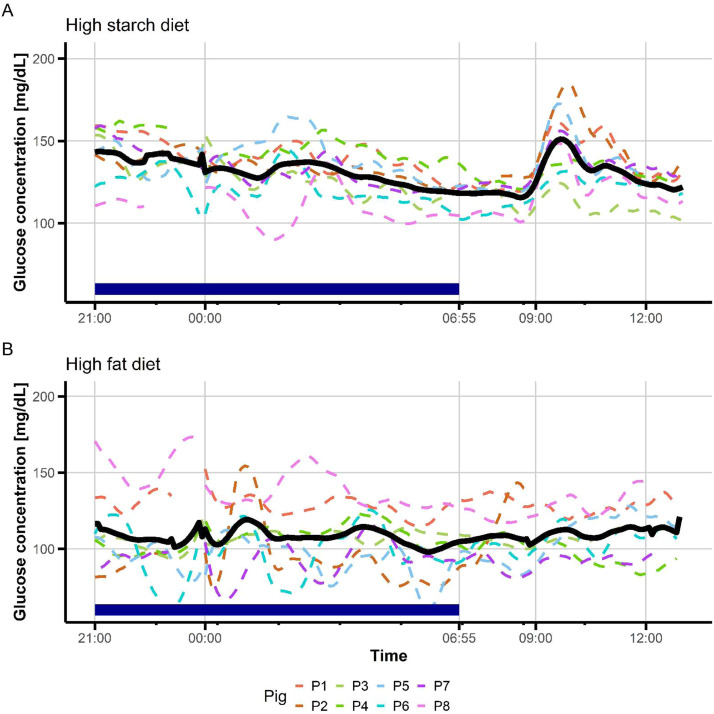
Overview of glucose wavelets during night and in postprandial period in growing pigs depending of the diet composition. Two meal test procedures (meal distribution at 0900 h) were conducted either with a high starch meal (A – HS, *n* = 6 pigs) or a high-fat meal (B – HF, *n* = 7 pigs). During the preceding night, pigs have no access to food. Glucose values were read every 5 min from a continuous glucose monitoring (CGM) sensor, and computed for the night and the period starting 15 min before and lasting for 3 h following the distribution of the morning meal test.

On the night preceding each meal test procedure, pigs were prevented from accessing feed from 1700 h. However, both peaks and nadirs of glucose concentrations were observed overnight (Fig. 2). The diet composition had a greater influence on nocturnal glucose excursions than it did on diurnal ones (Table 2). The number of nocturnal peaks tended to be 1.5-fold lower (1.5 peaks vs 2.3 peaks; *P*< 0.10) in the HS situation than in the HF situation, but the sharpest nocturnal peak lasted 1.8 times longer in the HS situation than in the HF situation (*P*< 0.01). The minimal glucose concentration in the night tended to be 1.3-fold higher (112 mg/dL vs. 84 mg/dL; *P*= 0.06) but the duration of the most pronounced nadir tended to be 1.9 times longer (70 min vs. 37 min; *P*= 0.07) in the HS situation than in the HF situation. Finally, the difference between minimal and maximal glucose concentrations during the night was 1.5-fold lower (27 % ± 5 % vs 41 % ± 12 %, *P*= 0.02) in the HS situation than in the HF situation. Overall, pigs were on euglycemic condition during approximately 73 % of the night time. Irrespective of the diet, glucose values above the baseline represented 13 % ± 4 % of the night time, whereas glucose values below the baseline represented 14 % ± 5 % of the night time.

### Correlations between nocturnal and postprandial glycemic excursions

Table 3 shows the CGM metrics that were (or tended to be) correlated (*P*< 0.10) between night 1 and postprandial morning period on d 2 for the HS diet (*n*= 6 pigs). The maximum glucose concentration during the postprandial diurnal period tended to be positively correlated with the duration of the most pronounced nocturnal glycemic peak and with the AUC of the most pronounced nocturnal nadir (Table 3). The duration of the sharpest glucose peak in the morning was negatively correlated with the number of nadirs recorded in the preceding night. Total duration of the moments where glucose concentrations were significantly above the baseline after eating the HS morning meal was negatively correlated with total AUC of the glucose peaks during the preceding night. On the opposite, the number of nadirs (i.e., glucose concentrations below baseline) observed in the morning period after consuming the HS meal test was positively correlated with the maximum and minimum nocturnal glucose concentrations, total AUC of the nocturnal peaks, total AUC of the nocturnal nadirs, and number of nocturnal nadirs.

**Table 3:** Correlations between nocturnal and postprandial glycemic excursions under a high starch (HS) diet.

CGM metrics			
Postprandial	Night^1^	ρ	*P*-value
Maximum glucose concentration	AUC of the sharpest nadir	0.77	0.10
Maximum glucose concentration	Duration of the sharpest peak	0.83	0.06
Duration of the sharpest peak	Number of nadirs	−0.74	0.10
Total duration of peaks	Total AUC of peaks	−0.84	0.04
Number of nadirs	Maximum glucose concentration	0.80	0.05
Number of nadirs	Duration of the sharpest peak	0.83	0.04
Number of nadirs	Minimal glucose concentration	0.80	0.05
Number of nadirs	Total AUC of nadirs	0.93	0.01
Number of nadirs	AUC of the sharpest nadir	0.83	0.04

Abbreviations: AUC = area under the curve.

1 Spearman (ρ) correlations were calculated between the characteristics of peaks and nadirs in glycemic excursions during the first 3 h after eating a high starch (HS) morning meal (postprandial period), and those recorded during the preceding night while the pigs had no access to food. All pigs (*n* = 6) were equipped with a continuous glucose monitoring (CGM) sensor. Only metrics that are significantly (*P* ≤ 0.05) or tending to be (0.05 < *P* ≤ 0.10) correlated were listed. Duration (in min) was defined as the time interval during which glucose values remained above or below the baseline within successive serial data for peak or nadir, respectively. Glucose concentrations were expressed in mg/dL.

Table 4 shows the CGM metrics that were (or tended to be) correlated (*P*< 0.10) between night 4 and postprandial morning period on d 5 for the HF diet (*n*= 7 pigs). The number of peaks after eating the morning HF test meal was negatively correlated with the duration and the AUC of the sharpest nocturnal glucose peak, and with total duration and total AUC of the nocturnal moments when glucose concentrations were above the baseline. The AUC of the sharpest glucose peak after eating the HF morning meal was negatively correlated with the maximum glucose concentration reached during the night. Conversely, the minimal value of glucose concentration during postprandial period was positively correlated with the number of nocturnal glucose peaks. The number of nadirs in the postprandial period was positively correlated with the minimal glucose concentration of the preceding night, whereas the total duration of nadirs (i.e., periods where glucose concentrations were below the baseline) after eating the HF morning meal was positively correlated with the maximum glucose concentration reached during the preceding night.

**Table 4:** Correlations between nocturnal and postprandial glycemic excursions under a high fat (HF) diet.

CGM metrics			
Postprandial	Night^1^	ρ	*P*-value
Number of peaks	Total duration of the peaks	−0.67	0.10
Number of peaks	Duration of the sharpest peak	−0.96	<0.001
Number of peaks	Total AUC of the peaks	−0.77	0.04
Number of peaks	AUC of the sharpest peak	−0.95	<0.001
AUC of the sharpest peak	Maximum glucose concentration	−1.00	0.08
Minimal glucose concentration	Number of peaks	0.87	0.06
Number of nadirs	Minimal glucose concentration	0.85	0.02
Number of nadirs	AUC of the sharpest nadir	−0.85	0.02
Number of nadirs	Total AUC of nadirs	−0.85	0.02
Total duration of nadirs	Maximum glucose concentration	0.82	0.09
Total duration of nadirs	Minimal glucose concentration	0.82	0.09
Total duration of nadirs	AUC of the sharpest nadir	−0.82	0.09
Total duration of nadirs	Total AUC of nadirs	−0.82	0.09

Abbreviations: AUC = area under the curve.

1 Spearman (ρ) correlations were calculated between the characteristics of peaks and nadirs in glycemic excursions during the first 3 h after eating a high fat (HF) morning meal, and those recorded during the preceding night while the pigs had no access to food. All pigs (*n* = 7) were equipped with a continuous glucose monitoring (CGM) sensor. Only CGM metrics that are significantly (*P* ≤ 0.05) or tending to be (0.05 < *P* ≤ 0.10) correlated were listed. Duration (in min) was defined as the time interval during which glucose values remained above or below the baseline within successive serial data for peak or nadir, respectively. Glucose concentrations were expressed in mg/dL.

### Comparisons between night and day glycemic excursions

**Fig. 3:**
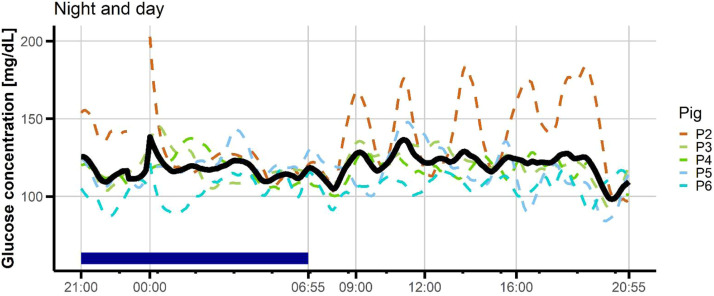
Overview of glucose wavelets overnight and the day in growing pigs fed high-starch based dietary formula. Glucose values were read every 5 min from a continuous glucose monitoring (CGM) sensor. Feed was removed at 1700 h and nocturnal events were computed from 2100 h to 0655 h. During the day after (0700 h-2055 h), pigs had free access to the food that was distributed twice in the day. The data were computed for night 2 and day 3 for pigs (*n* = 5) fed a 75 % high starch / 25 % high fat regimen.

To study the circadian rhythm of circulating glucose concentrations, we considered the night 2 and day 3 when pigs had free access to feed (no test meal procedure). At this time of the feeding trial, pigs were fed a 75 % HS and 25 % HF diet. Due to the difficulties mentioned above with the equipment, a complete dataset without any missing data was obtained from five experimental pigs (Fig. 3). The minimum glucose concentration tended to be lower (*P*< 0.10) in the day (89 mg/dL) than at night (100 mg/dL). Moreover, the AUC of the sharpest nadir in glucose concentrations at night tended to be lower than that observed in the day. There was no significant difference in the number of peaks and nadirs between night and day (Table 5). There was a greater inter-individual variability among pigs in the total duration and total AUC of peaks and nadirs during the diurnal period compared to the nocturnal period (*P*< 0.05). Conversely, there was less inter-individual variability among pigs for the AUC of the sharpest nadir at day than at night (*P*< 0.001).

**Table 5:** Description of glycemic excursions overnight and during the following day.

	Night^1^			Day				*P*-value	
CGM metrics	Mean	Min	Max	Mean	Min	Max	SEM	ANOVA^2^	F-test^3^
Peaks									
Number (n)	2.0	1.0	4.0	2.7	2.0	7.0	1.9	0.18	0.21
Total duration (min)	81	55	105	181	20	375	82	0.12	0.004
Total AUC	703	274	885	2248	18	8 218	2 165	0.32	<0.001
Maximum glucose concentration (mg/dL)	152	121	203	143	117	184	27	0.69	0.728
Duration of the most pronounced peak (min)	57	5	90	60	15	145	41	0.76	0.54
AUC of the most pronounced peak	621	29	736	710	16	1 640	593	0.62	0.06
Nadirs									
Number (n)	2.1	1.0	3.0	3.0	1.0	4.0	1.5	0.74	0.48
Total duration (min)	78	35	115	184	35	325	94	0.36	0.03
Total AUC	292	135	503	1 677	107	3 153	969	0.13	0.002
Minimum glucose concentration (mg/dL)	100	87	107	89	84	98	9	0.07	0.49
Duration of the most pronounced nadir (min)	51	35	110	91.4	20	155	46	0.51	0.39
AUC of the most pronounced nadir	213	85	281	1 084	64	1 853	619	0.08	0.001

Abbreviations: AUC = area under the curve.

1 Glucose excursions were monitored overnight and the following day, corresponding to night 2 and day 3 of the 5-days feeding trial. Pigs (*n* = 5) were fed a 75 % high starch / 25 % high fat mixed regimen during the day, and feed was removed at 1700 h. Nocturnal events were defined in the period from 21:00 to 06:55. The following day was defined as the period from 0700 h to 2055 h, and food was distributed twice the day. Duration was defined as the time interval during which glucose values remained above or below the baseline within successive serial data for peak or nadir, respectively.

2 ANOVA was used to compare means of the glycemic metrics between night and day.

3 F test was used to compare the variance between night and day.

**Fig. 4:**
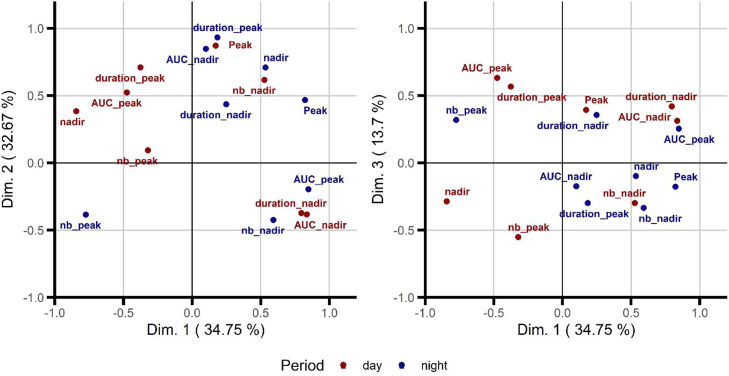
Clustering of glycemic characteristics over night and day in pigs.Principal component analysis (PCA) was used to represent the correlations between glycemic events identified during the night (blue dot) and the following day (red dot). Pigs (*n* = 5) were fed a 75 % high starch / 25 % high fat regimen. Feed was removed at 1700 h and the nocturnal events were computed from 2100 h to 0655 h. The day after (0700 h-2055 h), pigs had free access to the feed that was distributed ad libitum twice in the day. The values here referred to night 2 and day 3 of the 5-days feeding trial. AUC: area under the curve; duration: time interval (in min) during which glucose values remained above or below the baseline within successive serial data for peak or nadir, respectively; nb: number of peaks.

Finally, the CGM metrics for night and day were visualized on the principal component plot (PCA, Fig. 4). The upper right quarter gathered most of the diurnal glycemic metrics (except the number of peaks), whereas nocturnal glycemic metrics were distributed across the plot. Numbers of peaks during night and the day tended to be positively correlated (ρ = 0.80; *P*= 0.10). The maximum glucose concentrations during the night and the day also tended to be positively correlated (ρ = 0.90; *P*= 0.08).

## Discussion

The first challenge was to obtain complete CGM readings in free moving pigs for consecutive days and nights. It has been previously observed that vigorous contact against cage surfaces could shorten the device's lifespan and inquisitive cage-mates pose a significant threat to the device (Ober & Geist, 2020). We overcome this challenge for five pigs (day-night comparison) to seven pigs (night-morning postprandial analyses). In a similar pig genotype, Nadia et al. (2023) indicated that four pigs per diet are required to detect (power of 80 % and α of 0.05) a minimum difference of 900 mg/dL in AUC of plasma glucose and SD of 1 100 mg/dL. Lal et al. (2021) used only three pigs for documenting night and diurnal events in situation of streptozotocin‐induced insulin‐deficient diabetes, and Ober and Geist (2020) used four pigs to prove the capacity of CGM sensors to contribute to diabetic management. The natural chewing behavior of young, growing pigs clearly reduces the lifespan of the sensor, especially for animals housed in collective pens. This may require a compact bandage with a repulsive odor to secure the sensor, or a customized, tight harness adapted to the growth stage. From time-matched data obtained during two postprandial periods, we confirmed significant correlations between CGM readings and plasma glucose concentrations. All studies in pigs and miniature pigs have reported good correlations between CGM readings and glucose concentrations analyzed from blood samples, although the CGM demonstrated a greater bias than did glucometer measurements (Burnett et al., 1994; Strauss et al., 2008; Ober & Geist, 2020). The CGM sensors over-estimated the glucose concentrations compared to plasma values, a feature reported also in hyperglycemic pigs (Kvist et al., 2006) and streptozotocin‐induced diabetic pigs (Ober & Geist, 2020; Lal et al., 2021). Bias between CGM readings and blood glucose concentrations is explained by the fact that porcine red blood cell membranes are impermeable to glucose (Watts et al., 1979). The delay between CGM data compared to plasma data in the peak onset of glucose concentrations after eating the first morning meal, was similar to that reported by Malik (2022) in horses (∼12 min). This corresponds to an equilibration time between circulating blood at the jugular location and interstitial fluid collected at the neck (dorsal) subcutaneous location, so that interstitial glucose concentrations decreased before the plasma glucose concentrations in hypoglycemic situations and increased after plasma glucose concentrations in hyperglycemic situations (Malik, 2022). The correlation was however lower under HF diet than under HS diet. A HF meal can cause blood glucose levels to rise more slowly, starting several hours after eating and potentially lasting longer. It can also increase glucose production by the liver. Furthermore, a lower amplitude of glycemic excursions was observed in pigs fed the HF diet as compared to the HS diet. This may alter the correlation between CGM readings and glucose measurements from catheter blood samples.

To characterize glycemic excursions measured by CGM sensors, we defined a baseline for each pig as the mean glucose concentration (± SD) during the night before each day of the meal test procedure. In humans, MAGE captures also the average magnitude of significant glucose swings, both increases and decreases, by focusing on excursions that exceed one SD from the mean (e.g., Köhlmoos & Dittmar 2025). In studies with pigs fitted with an indwelling catheter, there was no other possibility than defining the initial time point just before eating the meal test as the base (Montagne et al., 2014; Le Floc'h et al., 2019), and intra- and inter-individual variations in this base are not considered (Eugenio et al., 2022). When analyzing CGM readings in pigs under clamp experiments (Aberer et al., 2020) or with streptozotocin-induced diabetes (Lal et al., 2021), threshold values have been considered with glucose concentrations <70 mg/dL for hypoglycemia and ≥180 mg/dL for hyperglycemia, respectively. In addition, Cichosz et al. (2025) recently stated that classical metrics such as time-in-range must be considered as distinct entities when analyzed from diurnal and nocturnal periods. In our study, pigs can be considered on a euglycemic situation during approximately 75 % of the night time. Lal et al. (2021) documented an overall time-in-euglycemic range of 90 % overnight in miniature and conventional pigs. However, they defined the night period as 0100 h–0700 h, whereas in our study, night was defined as 2100 h–0655 h based on the time at which the lights were switched off. As expected, CGM readings enabled postprandial changes in glucose concentrations to be followed and the effects of dietary composition on growing pigs to be deciphered. The CGM sensors detected a first peak of glucose between 35 and 155 min after eating the test meal, reflecting inter-individual variability among pigs in degrading, absorbing and using nutrients provided by diets with different forms of energy. Similarly, in pigs equipped with a central venous catheter, the onset of the postprandial glucose peak varied between 20 and 100 min after eating (Schop et al., 2023). In humans, high-fat diets, and especially those rich in saturated fats, typically led to lower glycemic variability, which results in lower MAGE and CONGA scores for high fat compared to high carbohydrate diets (Köhlmoos & Dittmar 2025). In our study, there were no significant differences in MAGE and CONGA measurements between diets. This may be due to the fact that HF diet was distributed in a shorter period (one day) compared to the extended period (several days) over which the HS diet was supplied. The composition of diet significantly influenced the maximum glucose concentration, with higher CGM values at peak for the HS diet than for the HF diet. This was expected due to the rapid release of glucose from starch in pigs fed a HS diet, whereas lipid supplementation in the HF diet spares glucose from catabolism. Other CGM metrics in postprandial period were similar between the two diets. These results are consistent with studies from catheter-based plasma glucose measurements, showing that pigs fed a diet containing five times the fat of a standard diet displayed no change in basal serum glucose concentrations (Charepalli et al., 2017). Similarly, dietary lipid supplementation in young pigs did not affect pre-prandial plasma glucose concentrations nor the rate at which plasma glucose increased after the meal (El-Kadi et al., 2019). Throughout the day, two to four peaks of glucose concentrations were observed based on the CGM readings, which is consistent with the biphasic behavior of the glycemic response curve in growing pigs eating a starch-based diet (Nadia et al., 2023).

An international consensus group on CGM metrics recommended analyzing glucose metrics by day and night blocks (Battelino et al., 2019). In our study, growing pigs exhibited glycemic peaks at night, which was not entirely expected given that they had no access to food overnight. Pigs were housed on slatted floors, which prevents them from inedible materials and limits the access to feces, even if no apparent excrement intake had been reported for pigs at 16 weeks of age (De Passillé et al., 1989). In healthy humans, nocturnal glucose excursions also showed transient hypoglycemia and hyperglycemia ranges (Köhlmoos & Dittmar 2025). Inter-individual variability among pigs in total duration and total AUC of peaks and nadirs was lower during the nocturnal period compared to the diurnal period. This is in accordance with data in humans showing that CV night-time was lower than CV day-time (Cichoz et al., 2025; Köhlmoos & Dittmar 2025). In mice, the consumption of a high-fat diet may advance the phase of the liver molecular clock and alters daily rhythms of eating behavior and locomotor activity (Branecky et al., 2015). In this study considering growing pigs, the number of nocturnal glycemic peaks tended to be lower in the HS than in the HF situation, and the difference between minimum and maximum glucose concentrations during the night was also less pronounced in the HS than in the HF situation. In pigs, meal intake in the late afternoon is generally greater than that in the morning (Boumans et al., 2017; Poullet et al., 2022), which could influence the pattern of nocturnal glycemic excursions. The data obtained herein support the view that a HF diet promotes long-term energy storage as lipids, which can be remobilized from the tissues in the form of non-esterified fatty acids (NEFA) during the nocturnal fasting period. Similarly, a HF diet in obese rats was associated with nocturnal hyperglycemia (Cano et al., 2008) and elevated NEFA at night acted as a signal for compensatory hyperinsulinemia in insulin-resistant dogs (Broussard et al., 2015).

Finally, we suggest that night-time glycemic excursions can affect postprandial responses to meal intake in the following morning. For instance, the maximal and minimal glucose concentrations during the night were positively related to the number of glycemic nadirs after having consumed the HS meal on the following morning. This suggests that larger fluctuations in glucose concentrations during the night led to faster glucose clearance during the day. Similarly, the carbohydrate intake (but not the carbohydrate/fat ratio) correlated positively with glycemic variability in healthy humans (Köhlmoos & Dittmar 2025). In our study, a higher number of nocturnal nadirs was associated with a shorter glycemic peak on the following morning when pigs consumed the HS meal. In humans, fasting glucose CGM readings in the morning were rather lower after nights with nocturnal hypoglycemia than after nights without hypoglycemia (Huang et al., 2022). The variations of nocturnal events, such as sleeping brainwaves or sleep perturbations, may influence glycemia regulation and insulin sensitivity in the day (Scheen et al., 1996; Vallat et al., 2023), with an increased AUC of glucose changes during the day observed after a perturbated night (Thiesse et al., 2018). It is difficult to translate this situation reported in humans who have variations in individual sleep-wake times, to growing pigs that continue drinking and have exploring activities for about 11–20 % of their night time (Meunier-Salaün et al., 2014). In human adults with type 1 diabetes, Vallat et al. (2023) indicated that both night-time and day-time glucose levels may contribute to long-term glycemic control. In our study, the relationships between nocturnal and diurnal CGM metrics remain complex, and some of them were even opposite according to the type of diet. For instance, total AUC of nocturnal glucose nadirs and the number of nadirs in the postprandial period were positively correlated for the HS diet but negatively correlated for the HF diet, respectively. The underlying mechanisms of the relationships between characteristics of glycemic excursions at night and energy regulation during the day remain to be elucidated. To achieve this, further projects should make use of video recording and an AI-assisted system to classify behaviors and link them to physiological or metabolic disturbances.

To conclude, this study has some limitations, primarily due to the small number of pigs enrolled in the trial and greater-than-expected inter-individual variability in glucose metrics. However, since asynchronous availability of glucose and amino acids within a day may change metabolism and nutrient retention (van den Borne et al., 2007), it opens new perspective for precision feeding technologies with the aim to optimize the alignment of energy metabolism and day and night cycles.

## Ethics

The experiment was performed in compliance with EU directive 2010/63/EU for animal experiments and French legislation. The technical and scientific staff had individual accreditation from the French minister to experiment on living pigs. The methods used for animal experiment were reviewed and approved by the local Committee on Ethics in animal experimentation (CREA07, Rennes, France) and the animal experimentation was authorized by the French Ministry of Higher Education, Research and Innovation under number APAFiS #37815-2,022062808557165 v3.

## Declaration of generative AI and AI-assisted technologies in the writing process

During the preparation of this work, the authors sometimes used DeepL Write to perfect the spelling of some sentences. The authors reviewed and edited the content as needed and take full responsibility of the content of the publication and the writing.

## CRediT authorship contribution statement

**Caroline Xavier:** Writing – review & editing, Writing – original draft, Visualization, Validation, Formal analysis, Data curation. **Francis Amann Eugenio:** Writing – review & editing, Visualization, Validation, Formal analysis, Data curation. **Charles-Henri Malbert:** Writing – review & editing, Visualization, Software, Resources, Methodology, Data curation. **Catherine Ollagnier:** Writing – review & editing, Project administration, Methodology, Funding acquisition, Conceptualization. **Sophie Brajon:** Writing – review & editing, Investigation, Conceptualization. **Florence Gondret:** Writing – review & editing, Writing – original draft, Validation, Supervision, Project administration, Methodology, Investigation, Funding acquisition, Conceptualization.

## Declaration of competing interest

We declare no conflict of interest.

## Appendix Supplementary materials

Image, application 1

Image, application 2
