# Supplementary material for: A pilot study testing a continuous glucose monitoring sensor in lean growing pigs fed contrasting diets, to document nocturnal and diurnal glycemic excursions as well as their relationships
Source: Vet Anim Sci. 2026 Mar 5;32:100612. doi: 10.1016/j.vas.2026.100612 (PMC12993900; doi:10.1016/j.vas.2026.100612)
Supplement: Supplementary file 1 [file mmc1.zip › Supplementary Figure S1.pptx]

## Slide 1
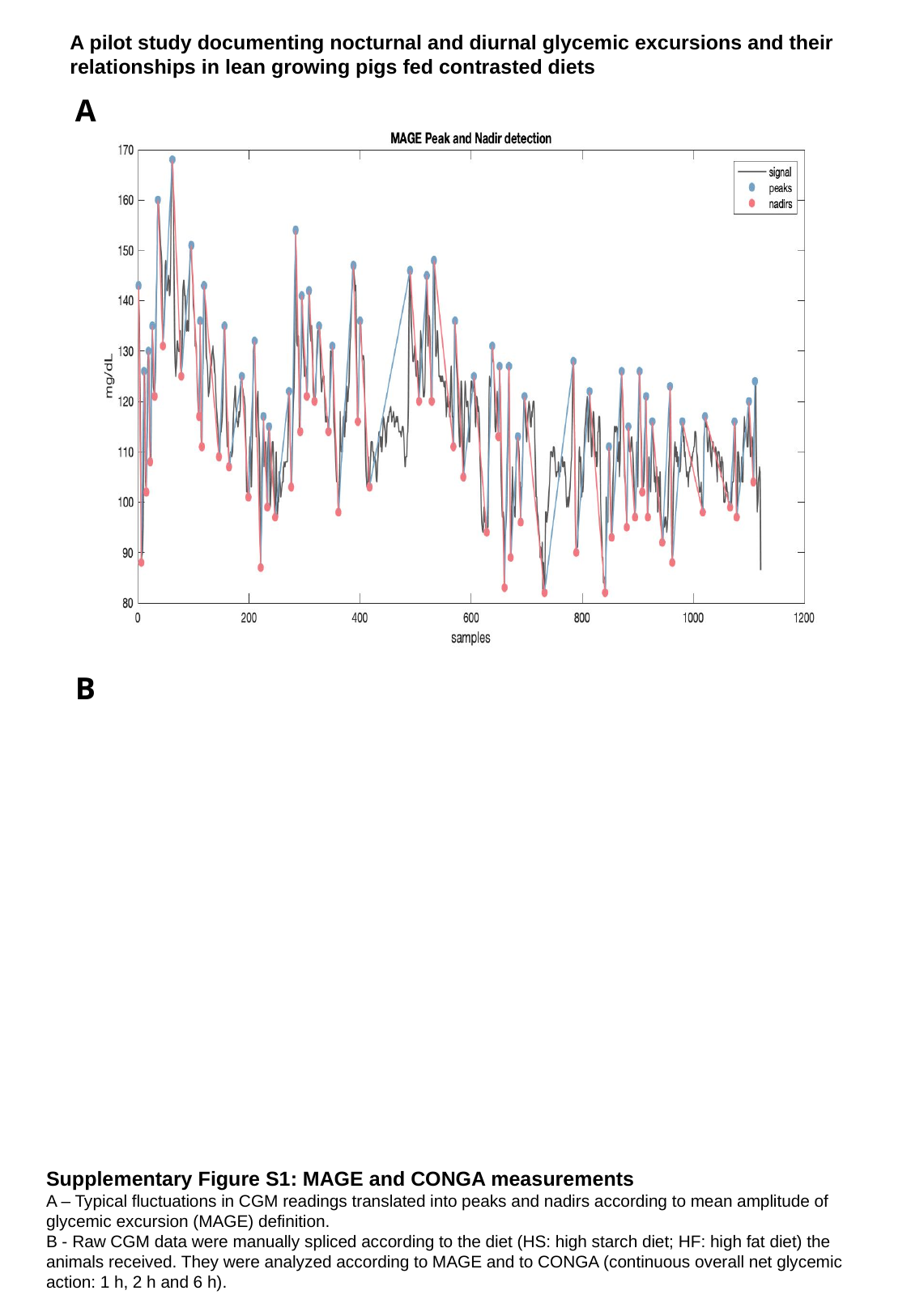

A pilot study documenting nocturnal and diurnal glycemic excursions and their relationships in lean growing pigs fed contrasted diets
A
B
Supplementary Figure S1: MAGE and CONGA measurements
A – Typical fluctuations in CGM readings translated into peaks and nadirs according to mean amplitude of glycemic excursion (MAGE) definition.
B - Raw CGM data were manually spliced according to the diet (HS: high starch diet; HF: high fat diet) the animals received. They were analyzed according to MAGE and to CONGA (continuous overall net glycemic action: 1 h, 2 h and 6 h).
