# Supplementary material for: A pilot study testing a continuous glucose monitoring sensor in lean growing pigs fed contrasting diets, to document nocturnal and diurnal glycemic excursions as well as their relationships
Source: Vet Anim Sci. 2026 Mar 5;32:100612. doi: 10.1016/j.vas.2026.100612 (PMC12993900; doi:10.1016/j.vas.2026.100612)
Supplement: Supplementary file 2 [file mmc2.zip › Supplementary Figure S2.pptx]

## Slide 1
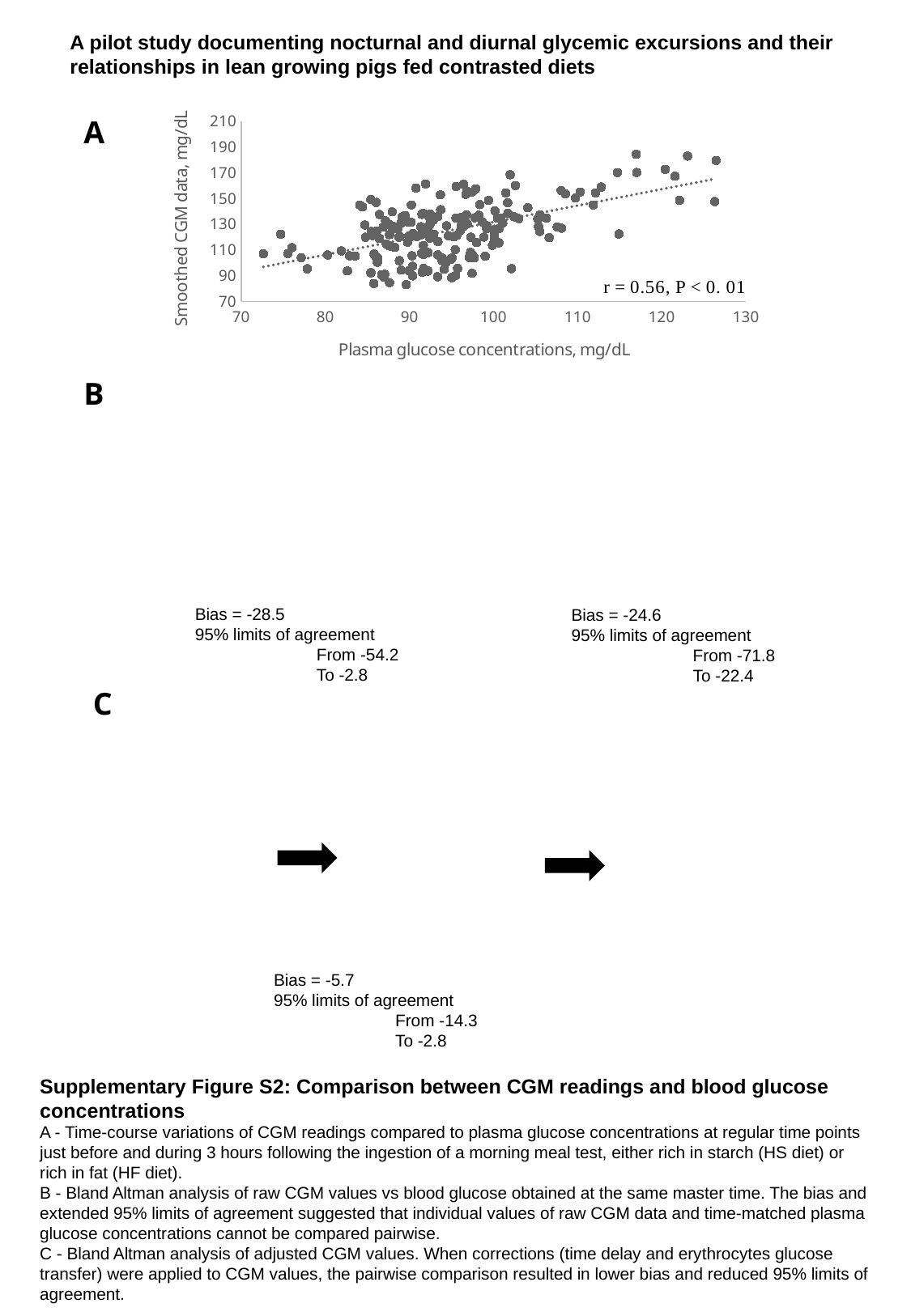

A pilot study documenting nocturnal and diurnal glycemic excursions and their relationships in lean growing pigs fed contrasted diets
### Chart
| Category | smoothed_sensor_glucose |
|---|---|A
B
Bias = -28.5
95% limits of agreement
	From -54.2
	To -2.8
Bias = -24.6
95% limits of agreement
	From -71.8
	To -22.4
C
Bias = -5.7
95% limits of agreement
	From -14.3
	To -2.8
Supplementary Figure S2: Comparison between CGM readings and blood glucose concentrations
A - Time-course variations of CGM readings compared to plasma glucose concentrations at regular time points just before and during 3 hours following the ingestion of a morning meal test, either rich in starch (HS diet) or rich in fat (HF diet).
B - Bland Altman analysis of raw CGM values vs blood glucose obtained at the same master time. The bias and extended 95% limits of agreement suggested that individual values of raw CGM data and time-matched plasma glucose concentrations cannot be compared pairwise.
C - Bland Altman analysis of adjusted CGM values. When corrections (time delay and erythrocytes glucose transfer) were applied to CGM values, the pairwise comparison resulted in lower bias and reduced 95% limits of agreement.
